# Antimicrobial Resistance Profiles of Bacteria Isolated from the Animal Health Sector in Zambia (2020–2024): Opportunities to Strengthen Antimicrobial Resistance Surveillance and Stewardship Programs

**DOI:** 10.3390/antibiotics14111102

**Published:** 2025-11-02

**Authors:** Taona Sinyawa, Fusya Goma, Chikwanda Chileshe, Ntombi B. Mudenda, Steward Mudenda, Amon Siame, Fred Mulako Simwinji, Mwendalubi Albert Hadunka, Bertha Chibwe, Kaunda Kaunda, Geoffrey Mainda, Bruno S. J. Phiri, Maisa Kasanga, Webrod Mufwambi, Samson Mukale, Andrew Bambala, Jimmy Hangoma, Nawa Mabuku, Benson Bowa, Obrian Kabunda, Mulumbi Nkamba, Ricky Chazya, Ruth Nakazwe, Mutila Malambo, Zoran Muhimba, Steven Mubamba, Morreah Champo, Mercy Mukuma, George Dautu, Chileshe Lukwesa, O-Tipo Shikanga, Freddie Masaninga, Mpela Chibi, Sandra Diana Mwadetsa, Theodora Savory, Joseph Yamweka Chizimu, John Bwalya Muma, Charles Maseka, Roma Chilengi

**Affiliations:** 1 Central Veterinary Research Institute, Ministry of Fisheries and Livestock, Chilanga, Lusaka 10101, Zambia; bowabenbig@yahoo.co.uk (B.B.); obkabunda@gmail.com (O.K.); lulunkamba@gmail.com (M.N.); gdautu@yahoo.co.uk (G.D.); 2 Department of Disease Control, School of Veterinary Medicine, University of Zambia, Lusaka 10101, Zambia; jmuma@unza.zm; 3 Department of Veterinary Services, Ministry of Fisheries and Livestock, Lusaka 15100, Zambia; fusya.goma@mfl.gov.zm (F.G.);; 4 Zambia National Public Health Institute, Stand 1186, Corner of Chaholi and Addis Ababa Roads, Rhodes Park, Lusaka 10101, Zambia; chikchile@gmail.com (C.C.); anita.kasanga@znphi.gov.zm (M.K.); mukalesamson14@gmail.com (S.M.); rchazya@yahoo.com (R.C.); zmuhimba@yahoo.com (Z.M.); mubambasteven8@gmail.com (S.M.); morreahchampo31@gmail.com (M.C.); clmusyami@yahoo.com (C.L.);; 5 Department of Biomedical Sciences, School of Veterinary Medicine, University of Zambia, Lusaka 10101, Zambia; 6 Department of Clinical Studies, University of Zambia, Lusaka 10101, Zambia; ntombi.nkonde@unza.zm; 7 Department of Pharmacy, University of Zambia, Lusaka 10101, Zambia; webrod.mufwambi@unza.zm; 8 Education and Continuous Professional Development Committee, Pharmaceutical Society of Zambia, Lusaka 10101, Zambia; 9 Centre for Research in Infectious Diseases, Lusaka 10101, Zambia; amon.siame@cidrz.org (A.S.); fred.simwinji@cidrz.org (F.M.S.); mwendalubi.hadunka@cidrz.org (M.A.H.); bertha.chibwe@cidrz.org (B.C.); kaunda.kaunda@cidrz.org (K.K.); theodora.savory@cidrz.org (T.S.); 10 Food and Agriculture Organization of the United Nations (FAO), Chaholi Road, Rhodes Park, Lusaka 10101, Zambia; geoffrey.mainda@fao.org; 11 Department of Paraclinical Studies, School of Veterinary Medicine, University of Zambia, Lusaka 10101, Zambia; bruno.phiri@unza.zm; 12 University Teaching Hospital, Ministry of Health, Lusaka 10101, Zambia; bambalaandrew@gmail.com (A.B.); ruthnakazwe@yahoo.com (R.N.); 13 Department of Pharmacy, School of Health Sciences, Levy Mwanawasa Medical University, Lusaka 10101, Zambia; jimmy.hangoma@lmmu.ac.zm; 14 Enhanced Smallholder Livestock Investment Project, Plot No. 1, Gizenga Road, Woodlands, Lusaka 10101, Zambia; 15 School of Agriculture, University of Zambia, Lusaka 10101, Zambia; mercy.mukuma@unza.zm; 16 Department of Health, World Health Organization, Lusaka 10101, Zambia; otipos@who.int (O.-T.S.); masaningaf@who.int (F.M.); mchibi@who.int (M.C.); mwadetsas@who.int (S.D.M.)

**Keywords:** antimicrobial resistance, antimicrobial stewardship, *Escherichia coli*, *Enterococcus* spp., *Salmonella* spp., *Klebsiella* spp., animal health, One Health, surveillance, Zambia

## Abstract

Background/Objectives: Antimicrobial resistance (AMR) is a major global health threat that undermines treatment in humans and animals. In Zambia, where livestock production underpins food security and livelihoods, AMR challenges are aggravated by limited surveillance, weak diagnostics, and poor regulatory enforcement, facilitating the spread of resistant pathogens across the human–animal–environment interface. This study aims to analyse AMR patterns of bacterial isolates collected from Zambia’s animal health sector between 2020 and 2024, to generate evidence that informs national AMR surveillance, supports antimicrobial stewardship (AMS) interventions, and strengthens One Health strategies to mitigate the spread of resistant pathogens. Methods: We conducted a retrospective descriptive analysis of previously collected routine laboratory data from five well-established animal health AMR surveillance sentinel sites between January 2020 and December 2024. Data were analysed by year, sample type, and antimicrobial susceptibility testing (AST) profiles using WHONET. Results: A total of 1688 samples were processed, with faecal samples accounting for 87.6%. Animal environmental samples (feed, manure, litter, abattoir/meat processing floor, wall, and equipment surface swabs) (collected from abattoirs, water, and farms) increased significantly over time (*p* = 0.027). Overall, *Escherichia coli* (*E. coli*) (50.4%) and *Enterococcus* spp. (30%) were the most frequently isolated bacteria. *E. coli* exhibited high resistance to tetracycline (74%) and ampicillin (72%) but remained susceptible to aztreonam (98%), nitrofurantoin (95%), and imipenem (93%). *Enterococcus* spp. were susceptible to penicillin (84%) and ampicillin (89%) but showed borderline resistance to vancomycin (53%) and linezolid (50%). *Klebsiella* spp. demonstrated resistance to ciprofloxacin (52%) and gentamicin (40%), whereas *Salmonella* spp. remained highly susceptible. Notably, resistance to amoxicillin/clavulanic acid rose sharply from 22.2% to 81.8% (*p* = 0.027). Across 1416 isolates, high levels of multidrug resistance (MDR) were observed, particularly in *E. coli* (48.4%) and *K. pneumoniae* (18.6%), with notable proportions progressing toward possible Extensively Drug-Resistant (XDR) and Pan-Drug-Resistant (PDR) states. Conclusions: The findings of this study reveal rising resistance to commonly used antibiotics in the animal health sector. Despite the lack of molecular analysis, our findings underscore the urgent need for AMS programs and integrated AMR surveillance under Zambia’s One Health strategy.

## 1. Introduction

Antimicrobial resistance (AMR), the failure of microorganisms to respond to antimicrobials to which they were once susceptible, is increasingly being recognised as a global public health and economic threat [1,2,3,4]. This phenomenon is currently referred to as the silent pandemic [3,5,6,7,8]. The overall health of humans, animals, and the environment is at dire risk, manifesting in compounded effects on food security and sustainable development [9,10]. Largely driven by the misuse and overuse of antimicrobials, livestock production systems are noted to be significant drivers of the rise and propagation of AMR [11]. The estimated global impact of AMR, if left unchecked, will lead to as high as 10 million deaths annually, with concomitant economic losses reaching USD 100 trillion by 2050 [12,13,14]. Evidence has shown an increase in antimicrobial use (AMU) in the animal sector, coupled with poor monitoring and surveillance, especially in most low- and middle-income countries (LMICs) [15,16,17,18,19,20]. This further results in the emergence and spread of AMR across the human–animal–environment continuum [21,22,23,24].

Drivers of AMR in the animal health sector have been reported in various studies [22,25,26,27]. The development of AMR in the animal health sector is largely due to the use of antimicrobials for therapy, metaphylaxis, prophylaxis, and growth promotion [28,29,30]. For example, evidence has indicated that the inappropriate use of antimicrobials in food-producing animals is a major driver of AMR [31,32]. This is coupled with using antimicrobials for growth promotion, increased production, and prophylaxis of diseases in the animal health sector [29,30,33,34]. Alongside this, access to antimicrobials without a valid prescription contributes to the development and spread of AMR [29,35,36,37,38]. Further, the lack of knowledge and awareness of AMU and AMR among farmers contributes to inappropriate use of antimicrobials and the emergence and spread of AMR [39,40,41]. Furthermore, veterinary antimicrobials contaminate the environment and expose microbes to these drugs, raising the potential for AMR emergence and spread [42]. Therefore, this calls for integrated AMR surveillance using a One Health approach because drug-resistant pathogens can cause infections in humans, animals, plants, and the environment [2,43,44,45].

Bacterial resistance to antibiotics is facilitated by a variety of mechanisms across bacterial genera [3,46,47,48]. The transfer of virulence and AMR genes across bacterial genera primarily occurs through horizontal gene transfer mechanisms such as conjugation, transformation, and transduction, often facilitated by mobile genetic elements like plasmids, transposons, and integrons [49]. These processes enable bacteria to rapidly acquire and disseminate resistance traits, even across unrelated species. Several factors accelerate this transmission, including excessive and inappropriate use of antimicrobials in livestock, intensive farming practices, inadequate biosecurity measures, and poor infection prevention and control [50,51,52]. Additionally, the close interaction between humans, animals, and the environment creates reservoirs that sustain resistant strains [53,54]. Together, these drivers intensify selective pressure, enhance bacterial adaptation, and promote the persistence and spread of AMR in animal health and beyond [22,55,56].

AMR surveillance reporting to global platforms like FAO InFARM has harmonised national AMR data with antimicrobial consumption (AMC) data via the ANImal antiMicrobial USE (ANIMUSE) WOAH global platform [57,58,59]. Effective surveillance, coupled with Antimicrobial Stewardship (AMS) programs, is an essential tool in mitigating the threat of AMR in the animal health sector [57,60,61]. As surveillance typically provides data on resistance patterns, it thus gives guidance on the formulation of treatment guidelines, policy development, and targeted interventions [45,62,63,64,65]. Stewardship programs promote rational AMU, as well as improved infection prevention practices, leading to enhanced overall quality of veterinary care [2,37,66,67,68,69,70,71].

Zambia, a country in the sub-Saharan African (SSA) region, developed a multisectoral National Action Plan (NAP) on AMR in 2017, having adapted it from the Global Action Plan on AMR [72]. This laid the foundation for an integrated AMR surveillance, key to which was the need for a well-coordinated, multi-pronged, One Health approach that integrated the human, animal, and environmental sectors as a result [73]. Arising from this, the animal health sector in Zambia selected *Escherichia coli* (*E. coli*)*, Enterococcus* spp. and *Salmonella* spp. as priority microorganisms, aligning with global target bacteria for AMR monitoring and surveillance in food-producing animals, in light of their relevance to public health [60].

There is evidence of AMR in the animal health sector in Zambia based on previous studies [25,71,74,75,76,77,78,79,80,81,82,83]. The burden of AMR in the Zambian animal health sector has been reported to be due to inadequate knowledge and awareness of AMR among farmers, lack of diagnostic capacity, access to antibiotics without prescriptions, and inadequate enforcement of laws that restrict the use of antimicrobials in animals [25,41,84,85]. However, there is a paucity of information regarding the AMR profiles of bacteria isolated from the animal health sentinel sites in Zambia. It is against this background that this study investigated the AMR profiles of bacteria isolated from the animal health sector in Zambia between 2020 and 2024.

By analysing trends in resistance, distribution of key pathogens, and patterns of antimicrobial susceptibility (AST), this study identifies key opportunities to strengthen AMR surveillance and AMS programs. The findings aim to support evidence-based decision-making and contribute to the ongoing implementation of the One Health approach to AMR containment in Zambia. Furthermore, the findings from this study are expected to guide the development of context-specific strategies, ensuring that policies and interventions are both effective and efficiently tailored to local conditions.

## 2. Results

### 2.1. Sample Distribution and Trends over the Five Years (2020–2024)

In Zambia, AMR surveillance data collection follows the integrated AMR surveillance framework [73]. Samples were processed in established AMR surveillance laboratories, including public laboratories (Central Veterinary Research Institute, and the Choma, Chipata, and Mongu Provincial Veterinary Diagnostic Laboratories), as well as academia (the University of Zambia Public Health Laboratory), before being tested for resistance and analysed using WHONET. The results showed that the majority of the samples processed between 2020 and 2024 were faecal at (*n* = 1478, 87.6%), indicating a strong focus on gastrointestinal zoonotic pathogen surveillance, as shown in Table 1. In the initial three years of animal health AMR surveillance, the focus was primarily on poultry cloacal swab/faecal samples, from which *E. coli* and *Salmonella* spp. were commonly isolated [86]. Faecal sample throughput declined marginally (*p* = 0.086), hinting at logistical delays or reduced sampling in animal health surveillance. Over the five years, the sample throughput did not increase significantly overall, except for animal environmental samples (feed, manure, litter, abattoir/meat processing floor, wall, and equipment surface swabs) (*n* = 169, 10%) that showed a significant increase, possibly reflecting heightened focus on biosecurity monitoring or environmental contamination surveillance. Meat samples (*n* = 26, 1.5%) and food samples (*n* = 15, 0.9%) were minimal, suggesting a limited emphasis on food safety or post-mortem diagnostics in this period.

### 2.2. Microbial Isolates Profile over Time (2020 to 2024)

**Figure 1:**
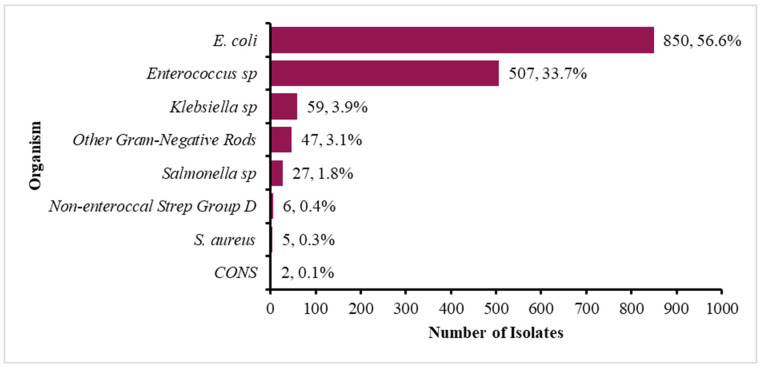
Profile of microorganisms isolated from different samples in animal health over the five years (2020 to 2024).

Figure 1 shows the frequency and percentage of the common isolates over the five years. *E. coli* (*n* = 850, 56.5%) and *Enterococcus* spp. (*n* = 507, 33.7%) were the most prevalent isolates, likely due to their ubiquity in zoonotic gut microbiota and role in AMR spread. Other pathogens included *Klebsiella* spp., (*n* = 59, 3.9%), *Salmonella* spp., (*n* = 27, 1.8%), and other Gram-negative rods, which included *Proteus* spp., *Citrobacter* spp., *Serratia* spp., and *Enterobacter* spp., culminating in a total of 47 (3.1%).

### 2.3. Isolation Rate of Pathogens by Sample Type

When split by sample type, Table 2 illustrates that *E. coli* (*n* = 754, 51%) and *Enterococcus* spp. (*n* = 498, 33.7%) were predominantly isolated in faecal samples such as cloacal swabs and faeces from poultry and other higher animals. Most of the *Klebsiella* spp. (*n* = 46, 27.2%), which included *K. pneumoniae*, *K. aerogenes*, and *K. oxytoca*, were isolated from animal environmental samples such as water, surface swabs, equipment, and animal pens, representing a significant faecal contamination of the environment. Furthermore, the isolation proportion of *Salmonella* spp. was relatively high in faecal (*n* = 19, 1.3%) and animal environmental samples (*n* = 7, 4.4%), with only one isolate (*n* = 1, 6.7%) from food samples like milk and eggs. Other Gram-negative rods were mostly isolated from faecal (*n* = 23, 1.6%) and animal environmental samples (*n* = 21, 12.4%).

### 2.4. Antibiotic Susceptibility Patterns of E. coli, Enterococcus spp., Klebsiella spp., and Salmonella spp.

**Figure 2:**
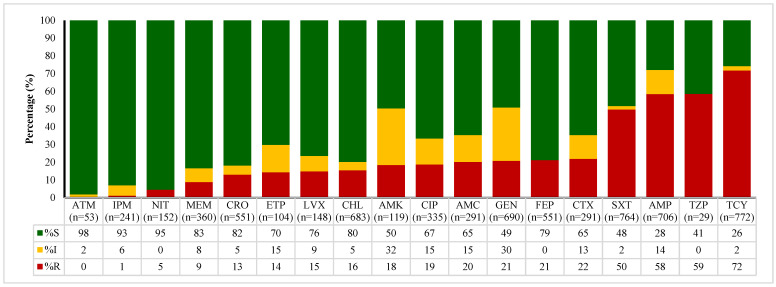
Antimicrobial susceptibility patterns of *E. coli* over time (2020 to 2024). ATM—aztreonam, IMP—imipenem, NIT—nitrofurantoin, MEM—meropenem, CRO—ceftriaxone, ETP—ertapenem, LVX—levofloxacin, CHL—chloramphenicol, AMK—amikacin, CIP—ciprofloxacin, AMC—amoxicillin—clavulanic acid, GEN—gentamicin, FEP—cefepime, CTX—cefotaxime, SXT—sulfamethoxazole/trimethoprim, AMP—ampicillin, TZP—piperacillin–tazobactam, TET—tetracycline. S—susceptible, I—intermediate, R—resistant.

**Figure 3:**
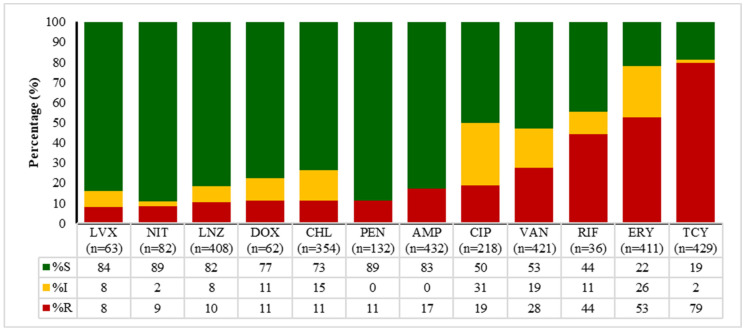
Antimicrobial susceptibility patterns of *Enterococcus* spp. Over time (2020 to 2024). LVX—levofloxacin, NIT—nitrofurantoin, LNZ—linezolid, DOX—doxycycline, CHL—chloramphenicol, PEN—penicillin, AMP—ampicillin, CIP—ciprofloxacin, VAN—vancomycin, RIF—rifampicin, ERY—erythromycin. S—susceptible, I—intermediate, R—resistant.

**Figure 4:**
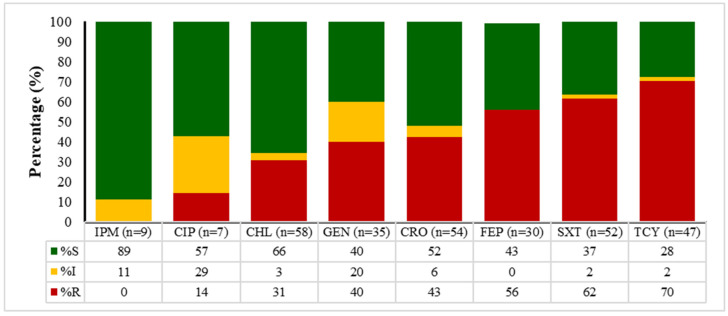
Antimicrobial susceptibility patterns of *Klebsiella* spp. over time (2020 to 2024). IMP—imipenem, CIP—ciprofloxacin, CHL—chloramphenicol, GEN—gentamicin, CRO—ceftriaxone, FEP—cefepime, SXT—sulfamethoxazole/trimethoprim, TET—tetracycline. S—susceptible, I—intermediate, R—resistant.

**Figure 5:**
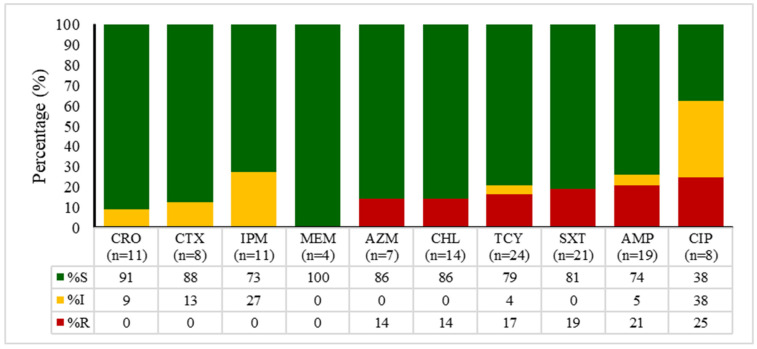
Antimicrobial susceptibility patterns of *Salmonella* spp. over time (2020 to 2024). CRO—ceftriaxone, CTX—cefotaxime, IMP—imipenem, MEM—meropenem, AZM—azithromycin, CHL—chloramphenicol, TET—tetracycline, SXT—sulfamethoxazole/trimethoprim, AMP—ampicillin, CIP—ciprofloxacin. S—susceptible, I—intermediate, R—resistant.

The data presented in Figure 2, Figure 3, Figure 4 and Figure 5 outline the antibiotic resistance patterns for various bacterial species, specifically *E. coli*, *Enterococcus* spp., *Klebsiella* spp., and *Salmonella* spp.

The resistance patterns for *E. coli* (Figure 2) show a wide variation across different antibiotics, with aztreonam, nitrofurantoin, and imipenem exhibiting high susceptibility rates (98%, 95%, and 93%, respectively), indicating that these antibiotics are generally effective against *E. coli*.

Ceftriaxone and meropenem have susceptibility rates of 82% and 83%, respectively, while ertapenem and levofloxacin showed slightly increased resistance with 70% and 76% susceptibility. Antibiotics that showed high resistance included tetracycline at 72%, with only 26% susceptibility, followed by ampicillin (28% susceptible) and piperacillin/tazobactam (41% susceptible).

For *Enterococcus* spp. (Figure 3), the resistance patterns also varied significantly, with penicillin G and ampicillin presenting with high susceptibility rates of 84% and 89%, respectively. Erythromycin also showed a good susceptibility rate of 89%. Linezolid and vancomycin show moderate susceptibility rates of 50% and 53%, respectively, indicating some level of borderline resistance in the animal population. Similar to the other isolates, tetracycline had a high resistance rate of 79%, with only 19% susceptibility, and doxycycline showed a similar trend.

*Klebsiella* spp. (Figure 4) displayed concerning resistance patterns, despite the number of isolates being few. Ceftriaxone was highly effective with 89% susceptibility, while imipenem showed 66% susceptibility. An increasing resistance was noted with gentamicin and ciprofloxacin, which showed significant resistance, with only 40% and 52% susceptibility, respectively. Tetracycline showed the highest resistance at 70%, with only 28% susceptibility.

*Salmonella* spp. (Figure 5) showed a generally favourable susceptibility profile with imipenem demonstrating complete susceptibility (100%), while ampicillin, ceftriaxone, and ciprofloxacin also demonstrated high susceptibility rates (91%, 88%, and 86%, respectively).

Meropenem and trimethoprim/sulfamethoxazole exhibited moderate susceptibility rates of 86% and 79%, respectively, while tetracycline showed significant resistance with only 38% susceptibility.

### 2.5. Trends of Antibiotic Non-Susceptibility over Time (2020–2024)

The results in Table 3 present the trends in antibiotic non-susceptibility from 2020 to 2024, including the results from the Mann–Kendall’s Tau test. The results showed that amoxicillin/clavulanic acid demonstrated a significant (*p* = 0.027) upward trend in non-susceptibility from 22.2% in 2020 to 81.8% in 2024, indicating an alarming resistance over time. On the other hand, ampicillin (AMP) displayed an upward trend from 51.1% to 71.9%, suggesting growing resistance, although the variation was not statistically significant (*p* = 1.000). Ciprofloxacin showed a fluctuating non-susceptibility, peaking at 57.1% in 2022 but dropping to 52.4% in 2024. Imipenem remained relatively stable but with low rates of non-susceptibility over the five years, indicating consistent susceptibility levels.

The proportion of multiple resistance patterns showed that multidrug resistance (MDR) was commonly demonstrated in *K. pneumoniae* isolates than in *E. coli* and *Enterococcus* sp.; however, there were very few (*n* = 23), as shown in Table 4.

## 3. Discussion

This study investigated the AMR profiles of bacteria isolated from the animal health sector in Zambia. This study found that between 2020 and 2024, AMR surveillance in Zambia’s animal health sector processed 1688 samples, predominantly faecal (87.6%), with a significant rise in animal environmental samples, reflecting increased biosecurity monitoring. *E. coli* (50.4%) and *Enterococcus* spp. (30.0%) were the most common isolates, while *Klebsiella* spp. and *Salmonella* spp. were more infrequently recovered from environmental sources. *E. coli* showed high susceptibility to aztreonam (98%), nitrofurantoin (95%), and imipenem (93%), but low susceptibility to tetracycline (26%) and ampicillin (28%). *Enterococcus* spp. were largely susceptible to ampicillin (89%) and penicillin (84%) but demonstrated borderline resistance to vancomycin (53%) and linezolid (50%). Notably, amoxicillin/clavulanic acid resistance increased sharply from 22.2% to 81.8% (*p* = 0.027), while carbapenem susceptibility remained high across all species. These findings highlight emerging resistance to commonly used antibiotics and the need for strengthened AMS and integrated One Health surveillance.

The overwhelming majority of samples analysed were faecal (87.6%), primarily from poultry and other livestock, reflecting a surveillance bias toward enteric zoonoses like *E. coli* and *Enterococcus* spp. This is consistent with AMR studies in sub-Saharan Africa, where food animals are routinely monitored for gastrointestinal pathogens due to their public health relevance and ease of sample collection [86,87,88]. Animal environmental samples increased significantly during the study period, suggesting an expanding interest in biosecurity and the environmental dissemination of resistance genes. Such improvements are key to preventing spillover between animals, humans, and ecosystems [89]. Conversely, the underrepresentation of food (0.9%) and meat (1.5%) samples highlights underutilised opportunities for food safety and post-mortem surveillance.

Our study found that *E. coli* (50.4%) and *Enterococcus* spp. (30.0%) were most frequently isolated, reflecting their ubiquity in gut microbiota and role in AMR spread. The isolation of *E. coli* and *Enterococcus* spp. from gut microbiota in animals in Zambia has been reported in earlier studies, especially in the poultry sector [74,75,76,90,91,92]. The presence of *Salmonella* spp. has been reported in the animal health sector in Zambia [78,81,93,94]. The present study found that *Klebsiella* spp. and *Salmonella* spp. were more often recovered from animal environmental samples, indicating possible faecal contamination pathways. Previous studies have also demonstrated the presence of *Klebsiella* spp. and *Salmonella* spp. from domestic animals, with potential for contamination [95,96,97,98,99,100,101]. The isolation of *E. coli*, *Enterococci* spp., *Salmonella* spp., and *Klebsiella* spp. in food-producing animals indicates the need to conduct surveillance using a One Health approach because these bacteria are also isolated in humans and the environment [2,71,102,103].

The present study found that *E. coli* exhibited high susceptibility to aztreonam (98%), nitrofurantoin (95%), and imipenem (93%), but low susceptibility to tetracycline (26%) and ampicillin (28%). The high susceptibility to carbapenems aligns with findings from previous studies conducted in Zambia [78,79,81,104]. This observation is likely attributable to the fact that carbapenems are not routinely used in the animal health sector in Zambia, thereby limiting the selective pressure for resistance development.

Our study revealed that *Enterococcus* spp. demonstrated high susceptibility to ampicillin (89%) and penicillin (84%) but showed borderline resistance to vancomycin (53%) and linezolid (50%). The present findings are in contrast with a previous study performed in Zambia, which found high resistance of enterococci to ampicillin, although similar resistance to linezolid was documented [76]. Evidence of vancomycin-resistant enterococci was also reported in Tanzania across human and animal samples, indicating a One Health problem implicated by this pathogen [105]. *Klebsiella* spp. exhibited good susceptibility to ceftriaxone (89%) and imipenem (66%), but elevated resistance to ciprofloxacin (52%) and gentamicin (40%). A study conducted in Zambia in the human health sector found high resistance of *K. pneumoniae* to third-generation cephalosporins such as ceftriaxone, indicating high use of cephalosporins in the human health sector compared to the animal health sector in Zambia [106]. A recent study conducted in Lusaka, Zambia, found that *K. pneumoniae* was highly susceptible to carbapenems, including ertapenem and imipenem [107]. On the other hand, *Salmonella* spp. displayed generally high susceptibility, particularly to imipenem (100%), ceftriaxone (88%), and ciprofloxacin (86%), although reduced susceptibility was observed for tetracycline (38%). Previous studies conducted in Zambia have also demonstrated high resistance of *Salmonella* spp. isolated from poultry to tetracycline [78,81]. This could be due to the high use of tetracycline in the poultry sector in Zambia, especially with access without veterinary prescriptions [25,35,41,84]. The observed resistance of bacteria to antibiotics in the animal health sector in Zambia calls for urgent solutions, including the initiation of AMS programs and other innovative strategies to combat the rising AMR rates [2,71,84,108].

Our study demonstrated a significant increase in non-susceptibility to amoxicillin/clavulanic acid, rising from 22.2% in 2020 to 81.8% in 2024 (*p* = 0.027). Alongside this, ampicillin resistance increased from 51.1% to 71.9% over the study period, although the change was not statistically significant. Further, ciprofloxacin resistance fluctuated but remained high in some years (>50%). Furthermore, carbapenems (imipenem, meropenem) maintained low resistance, indicating preserved efficacy. Therefore, our study demonstrated evidence of the rising resistance to commonly used antibiotics, which underscores the need for strict AMS in veterinary settings. The findings highlight the need to preserve carbapenems and other critically important antimicrobials, which is crucial to prevent cross-sectoral resistance spillover. Alongside this, there is a need for targeted interventions to reduce the overuse of tetracyclines and β-lactams in livestock. These findings indicate the need to establish and strengthen AMS programs in the animal health sector in Zambia, similar to the findings and recommendations from other studies [67,71,109].

Overall, of the 1416 isolates tested, (*n* = 482, 34.1%) were MDR, (*n* = 378, 26.7%) were possible XDR, and (*n* = 141, 10.0%) were possible PDR, with particularly high proportions in *E. coli* (42.9%) and *K. pneumoniae* (47.8%). The results are inconsistent with other studies in Africa that revealed higher MDR prevalence among several bacterial isolates from livestock [110,111,112,113]. Even though lower, these results underscore the widespread presence of resistance across livestock-associated bacteria and the narrowing spectrum of effective antimicrobials. The findings support the urgent need for enhanced AMS, prudent drug use policies in veterinary medicine, and continued genomic surveillance to better understand the mechanisms and drivers of resistance.

The findings of the present study also indicate the need to expand sampling beyond faecal to include more animal environmental, food, and meat samples for a holistic One Health view. Alongside this, there is a need to integrate molecular typing and resistance gene detection to track AMR transmission pathways. Additionally, there is a need to strengthen data reporting to platforms like FAO InFARM and WOAH ANIMUSE for global comparability. These findings demonstrate the need to strengthen AMR surveillance in animal health, similar to findings from previous studies [61]. There is also a need to integrate AMR surveillance across the One Health sector to provide comprehensive AMR surveillance data [54,114,115].

We are aware that this study has some limitations that should be considered when interpreting the findings. First, it was based on retrospective analysis of laboratory surveillance data from selected sentinel sites, which may not fully represent AMR patterns across all regions, livestock systems, and animal species in Zambia. Second, the sample distribution was heavily skewed towards faecal samples, with limited representation of animal environmental, meat, and food samples, potentially underestimating AMR prevalence in other important reservoirs. The imbalance in sample types and the lack of proportional sampling limit this study’s ability to claim national representativeness, and as such, the findings are primarily indicative of resistance patterns within the sampled sites and sample types. Third, the dataset was restricted to phenotypic AST, with no molecular characterisation of resistance genes or strain typing, limiting the ability to determine genetic mechanisms of resistance and potential transmission pathways. The absence of molecular data prevents the identification of specific resistance genes or a definitive understanding of gene transfer. Fourth, variations in sampling intensity, diagnostic capacity, and laboratory methodologies across sites and over time may have introduced inconsistencies and bias in the results. Fifth, this study could not directly correlate AMR patterns with AMU. Finally, due to the reliance on available surveillance data, some pathogens of potential public health significance may have been missed, and temporal changes in resistance could reflect shifts in sampling or testing priorities rather than true epidemiological trends. Therefore, we recommend the need for a comprehensive, integrated surveillance system that includes both AMR and AMU data to better inform policy and intervention strategies.

The findings from this study have several important policy implications for strengthening AMR control in Zambia’s animal health sector, as shown in Table 5. The current surveillance system, which is heavily dominated by faecal sampling, should be expanded to routinely include animal environmental, food, and meat samples to better capture AMR risks across the One Health interface. The observed increase in animal environmental sampling highlights the need to institutionalise this approach within national AMR surveillance guidelines, alongside stronger farm and slaughterhouse biosecurity measures to reduce animal environmental contamination by pathogens such as *Klebsiella* spp. and *Salmonella* spp. The continued focus on priority indicators like *E. coli* and *Enterococcus* spp. is essential, but should be complemented by monitoring other relevant pathogens to broaden risk assessment. The high resistance of *E. coli* to tetracycline and ampicillin, coupled with the alarming rise in amoxicillin/clavulanic acid resistance, underscores the urgency of regulating veterinary antimicrobial sales, restricting growth promoter use, and promoting culture-based prescriptions. Borderline resistance in enterococci to vancomycin and linezolid signals the need to protect critically important human medicines from veterinary misuse. Preserving carbapenem efficacy by maintaining them as last-resort drugs and introducing stewardship protocols for fluoroquinolone use are vital to slowing resistance spread. Finally, investing in molecular epidemiology capacity and integrating results into global reporting platforms will enhance detection of resistance mechanisms, support targeted interventions, and align Zambia’s surveillance with international AMR control efforts.

## 4. Materials and Methods

### 4.1. Study Design and Setting

**Figure 6:**
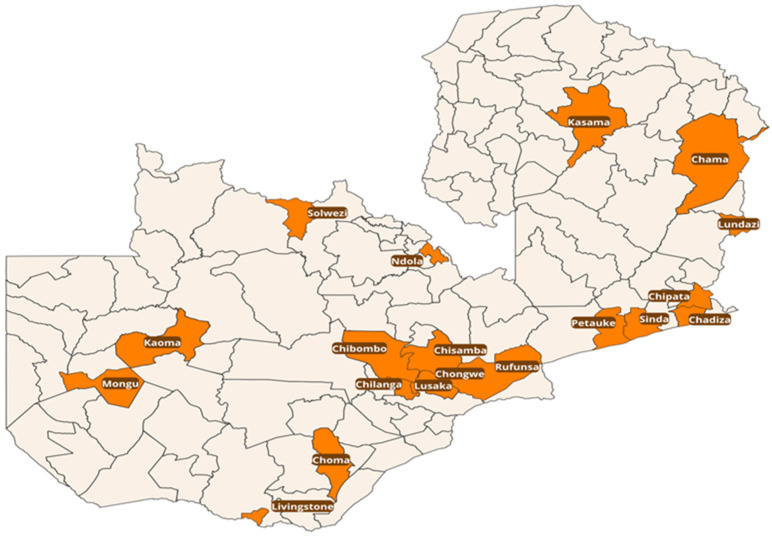
Map showing the districts from which samples were collected during the study period.

This retrospective study analysed routine laboratory data that were collected from January 2020 to December 2024 from four public and one academic animal health AMR surveillance sentinel sites across Zambia. The nationwide surveillance collects samples from all administrative provinces through a through a multistage stratified sampling technique down to districts and farms surrounding the five animal health laboratories: the Central Veterinary Laboratory Institute (CVRI) (Reference AMR laboratory), Choma Provincial Veterinary Laboratory, Chipata Provincial Veterinary Laboratory (CPVL), Mongu Provincial Veterinary Laboratory (MPVL), and the University of Zambia School of Veterinary Medicine Microbiology Laboratory (UNZA VET Lab), as shown in Figure 6. These were designated for National AMR surveillance following the FAO-ATLAS assessment that defined targets to improve national AMR surveillance systems in the food and agriculture sectors. The sentinel sites were selected based on their established capacity to perform AMR surveillance. To ensure reliable results and strengthen data validity despite routine diagnostic limitations, this study leveraged reference laboratories with advanced microbiological capacity, supported by mentorship and external quality assurance (EQA).

### 4.2. Study Design

Laboratory AMR surveillance data were collected retrospectively from 2020 to 2024. The samples were collected from poultry, cattle, and the animal environment. The laboratories tested samples from market-ready broiler and layer chickens (four weeks and above for broilers and at the point of lay for layers), cattle above 2 years, and environmental samples. At this stage of sampling, animals are towards the end of production, just before entering the food chain, and thus provide additional information on food safety.

### 4.3. Sample Type and Sample Size

This study included all five of the animal health AMR sentinel sites in Zambia. The laboratory network obtained various sample types, including cloacal swabs from poultry, faecal and milk samples from beef and dairy animals, as well as surface swabs from animal environments. The sample size for estimation for the poultry and beef samples was based on the national estimates as defined in the respective national surveillance protocols that stipulated the proportional distribution of the animal population.

### 4.4. Sampling Strategy

Sampling was conducted proportionally in each of the respective strata in the sampling frame, ensuring a representative distribution aligned with the overall population structure. This allowed for a better representation of the strata, depicted by the proportional share in the overall population. The surveillance considered the primary sampling unit to be a “farm”; in situations where the farm had more than one house or kraal, each one was an independent unit.

### 4.5. Data Collection

The data were obtained from previously isolated and identified samples, collected by trained laboratory personnel at the five sentinel sites under the supervision of the national AMR surveillance program. Sample processing was standardised across all sentinel sites, following established laboratory standard operating procedures (SOPs). The surveillance targeted organisms in healthy livestock, including *E. coli*, *Enterococcus* spp., *Klebsiella* spp., and *Salmonella* spp. These organisms had already been isolated and identified using standardised microbiological procedures, including culture and confirmation through biochemical testing [75,76,78,79,81,90,107]. All data for this compilation were derived from active surveillance of healthy, market-ready chickens (cloacal swabs, faecal samples, and eggs), cattle (faecal samples and milk), and their environments (feed, manure, litter, as well as abattoir and meat-processing surfaces, including floors, walls, and equipment). All data used for this compilation were obtained from active surveillance of healthy, market-ready chickens, cattle, and the animals’ environment. Organism selection was based on the priority pathogen list as described by the Global Antimicrobial Resistance and Use Surveillance System (GLASS) [116].

### 4.6. Antimicrobial Susceptibility Testing

The antibiotics used were selected according to the WHO model list of essential drugs and commonly used antibiotics in Zambia [117]. The antimicrobial classes tested included tetracyclines, penicillins, sulphonamides, macrolides, quinolones, cephalosporins, chloramphenicol, monobactams, aminoglycosides, rifamycin, nitrofurans, *β*-lactam inhibitors, and carbapenems. The AST testing was performed using the Kirby–Bauer disc diffusion method. The AST results were entered directly into the WHONET database, with the inbuilt Clinical Laboratory Standards Institute (CLSI) guidelines M100 used for the interpretation of AST results [118].

### 4.7. Quality Control and Inter-Laboratory Comparability

To ensure reliability and accuracy, quality control was carried out on media, reagents, and antibiotic disks using standard ATCC reference strains (*E. coli* ATCC 25922, *P. aeruginosa* ATCC 27853, *K. pneumoniae* ATCC 700603, *S. aureus* ATCC 25923, *E. faecalis* ATCC 29212). All sentinel sites followed harmonised SOPs, with results interpreted using CLSI guidelines and standardised in WHONET. Inter-laboratory comparability was maintained through participation in EQA and periodic re-testing at the national reference laboratory.

### 4.8. Data Analysis

Descriptive statistics were performed using WHONET 2025 and IBM SPSS version 25.0. The WHONET database was used to collect and analyse AST data, including test results of isolates, metadata, and AMR profiles. This study concentrated on comparing the rates of AMR in the bacterial isolates, determining which antibiotics are most frequently ineffective, analysing the frequency of resistance to different antibiotics, and monitoring trends over time. AMR profiles were reviewed over the five years (2020–2024), and data were visualised using charts and tables. Confidence ranges for statistical comparisons of resistance rates, including AST graphs, tables and charts, were provided by IBM SPSS version 25.0 to illustrate the resistance patterns of bacteria to different antibiotics and to ensure a clear presentation of the data.

## 5. Conclusions

This five-year surveillance analysis highlights a significant but addressable AMR burden in Zambia’s animal health sector. The predominance of *E. coli* and *Enterococcus* spp., combined with rising resistance to widely used antibiotics such as tetracycline and ampicillin, signals a pressing threat to animal and public health. Although susceptibility to last-resort agents like imipenem and aztreonam remains largely preserved, the notable resistance observed in pathogens such as *Klebsiella* spp. and *Salmonella* spp. underscores the urgency of regulating and rationalising AMU in livestock. This study also reveals critical surveillance gaps, including underrepresentation of animal environmental, meat, and food samples, pointing to the need for broader sample coverage. The findings provide opportunities for strengthening surveillance and stewardship programs, while further studies are needed to confirm the AMR trends and to link them to specific interventions. Embedding these measures within a comprehensive One Health framework offers the best pathway to protecting the efficacy of life-saving antibiotics and safeguarding health across human, animal, and environmental domains.

## Figures and Tables

**Table 1:** Total samples cultured between 2020 and 2024.

Sample Type	Total	2020	2021	2022	2023	2024	*p* Value *
	*n* (%)	*n* (%)	*n* (%)	*n* (%)	*n* (%)	*n* (%)	
Faecal	1478 (87.6%)	524 (98.5%)	336 (92.0%)	219 (92.0%)	255 (85.6%)	144 (56.5%)	0.086
Animal environmental	169 (10.0%)	0 (0%)	5 (1.4%)	18 (7.6%)	41 (13.8%)	105 (41.2%)	0.027
Meat	26 (1.5%)	0 (0%)	24 (6.6%)	0 (0%)	2 (0.6%)	0 (0%)	1.000
Food	15 (0.9%)	8 (1.5%)	0 (0%)	1 (0.4%)	0 (0%)	6 (2.3%)	1.000
Total	1688	532	365	238	298	255	0.086

* Mann–Kendall Tau’s *p*-value for trends over five years. Note: Some sample types recorded zero in 2020 and 2021, possibly due to COVID-19, which caused a reduction in sample throughput.

**Table 2:** Bacterial Isolate Profiles by Sample Type.

Organism	Faecal (*n* = 1478)		Animal Environmental (*n* = 169)		Meat (*n* = 26)		Food (*n* = 15)		Total (*n* = 1688)	
	*n*	%	*n*	%	*n*	%	*n*	%	*n*	%
*Escherichia coli*	754	51.0%	77	45.6%	8	30.8%	11	73.3%	850	50.4%
*Enterococcus* spp.	498	33.7%	6	3.6%	3	11.5%	0	0.0%	507	30.0%
*Klebsiella* spp.	13	0.9%	46	27.2%	0	0.0%	0	0.0%	59	3.5%
*Other GNRs*	23	1.6%	21	12.4%	2	7.7%	1	6.7%	47	2.8%
*Salmonella* spp.	19	1.3%	7	4.1%	0	0.0%	1	6.7%	27	1.6%
Non-*enterococcal Strep* Group D	6	0.4%	0	0.0%	0	0.0%	0	0.0%	6	0.4%
*Staphylococcus aureus*	1	0.1%	0	0.0%	2	7.7%	2	13.3%	5	0.3%
CONS	0	0.0%	0	0.0%	2	7.7%	0	0.0%	2	0.1%

**Table 3:** Trends of antibiotic non-susceptibility over time (2020–2024) and the Mann–Kendall Tau’s test of trends.

Antibiotics	Period: 2020–2024					Mann–Kendall’s Tau Test		
	2020	2021	2022	2023	2024			
	%NS	%NS	%NS	%NS	%NS	Kendall’s *Tau*	*p-Value*	*Sen’s Slope*
AMC %NS	22.2	26.8	36.9	48.8	81.8	1.000	0.027	11.450
AMP %NS	51.1	51.2	49	44.7	71.9	0.000	1.000	−0.475
CHL %NS	23.3	17.9	21.2	27.7	25.5	0.400	0.462	1.808
CIP %NS	35.7	25.7	57.1	40.5	52.4	0.400	0.462	5.788
FEP %NS	0.6	5.2	14.3	7.7	28.3	0.800	0.086	6.888
CTX %NS	31.3	20.9	44.4	87.5	35.5	0.400	0.462	5.708
CRO %NS	7.5	8.3	25.5	14	48.8	0.800	0.086	9.663
CAZ %NS	5.5	2.6	22.7	29.4	44.1	0.800	0.086	10.175
ERY %NS	86.5	75.2	86.6	68.3	76.7	−0.200	0.806	−2.950
GEN %NS	44	49	58	40.5	42.5	−0.200	0.806	−0.771
IPM %NS	4.3	17.9	9.1	9.2	10.5	0.400	0.462	1.000
LNZ %NS	28.1	20.5	35.4	23.2	20.8	−0.200	0.806	−1.729
MEM %NS	0	10.1	33.3	20.3	39.1	0.800	0.086	9.721
TCY %NS	80.9	72.1	79.5	59.7	79.9	−0.200	0.806	−0.475
SXT %NS	50	52.7	45.2	48.1	56.2	0.200	0.806	1.358
VAN %NS	45.1	35.3	83.5	38.6	81.8	0.200	0.806	5.413
LVX %NS	0	15.7	22.1	76.9	10	0.400	0.462	8.725
AMK %NS	30.8	0	100	73.8	60	0.200	0.806	10.817

**Table 4:** Proportion of multidrug resistance patterns of *E. coli*, *Enterococcus* spp. and *Klebsiella* spp. isolated from animal health sources.

Organism	Number of Isolates	MDR	Possible XDR	Possible PDR
*E. coli*	850	411 (48.4%)	313 (36.8%)	110 (12.9%)
*Enterococcus* spp.	507	60 (11.8%)	54 (10.7%)	21 (4.1%)
*Klebsiella* spp.	59	11 (18.6%)	11 (18.6%)	10 (16.9%)
Total	1416	482	378	141

**Table 5:** Policy implications of AMR surveillance findings in Zambia’s animal health sector (2020–2024).

Key Finding	Policy Implication	Proposed Action	Responsible Stakeholders
High proportion of faecal samples (87.6%) and limited animal environmental, food, and meat sampling	Surveillance scope is narrow, potentially missing AMR sources in the animal environment and food chain	Expand sentinel site protocols to include routine animal environmental, meat, and food sample collection to support One Health surveillance	Ministry of Fisheries and Livestock (MFL); Zambia National Public Health Institute (ZNPHI); Ministry of Health (MoH); FAO
Significant increase in animal environmental samples (*p* = 0.027)	Growing recognition of animal environmental AMR risks	Institutionalise animal environmental sampling in AMR surveillance guidelines and integrate with environmental health monitoring	MFL; ZNPHI; Zambia Environmental Management Agency (ZEMA)
*E. coli* and *Enterococcus* spp. dominate isolates	These pathogens are priority AMR indicators and can spread resistance genes	Maintain focus on these species while adding other relevant pathogens for comprehensive risk profiling	MFL; MoH; FAO; WOAH
*Klebsiella* spp. and *Salmonella* spp. are more prevalent in environmental samples	Environmental contamination may be a significant reservoir for resistant pathogens	Strengthen farm-level and slaughterhouse biosecurity measures; enforce waste management standards	MFL; Local Authorities; ZEMA; Food Safety Agencies
Low susceptibility of *E. coli* to tetracycline (26%) and ampicillin (28%)	Overuse of common antimicrobials in veterinary practice is likely contributing to resistance	Regulate veterinary antimicrobial sales; implement restrictions on growth-promoter use; encourage alternatives to antibiotics	Veterinary Council of Zambia (VCZ); MFL; Zambia Medicines Regulatory Authority (ZAMRA)
Borderline resistance in *Enterococcus* spp. to vancomycin (53%) and linezolid (50%)	Potential emergence of resistance to critically important human medicines	Enforce strict controls on veterinary use of critical antimicrobials; introduce a national “protected list” of antibiotics	MoH; MFL; ZAMRA; VCZ
Increasing resistance to amoxicillin/clavulanic acid (22.2% → 81.8%, *p* = 0.027)	Rapid resistance escalation to a key broad-spectrum antibiotic	Review and restrict empirical veterinary use of amoxicillin/clavulanic acid; promote targeted therapy based on susceptibility testing	MFL; VCZ; ZAMRA
Preserved susceptibility to carbapenems (imipenem, meropenem)	Critical antimicrobials remain effective	Maintain carbapenems as “last-resort” drugs; ban routine veterinary use to prevent resistance development	MoH; MFL; ZAMRA
Fluctuating ciprofloxacin resistance (peaking > 50%)	Risk to human medicine, as fluoroquinolones are important in both sectors	Introduce stewardship protocols for fluoroquinolone use in livestock; require culture and sensitivity testing before administration	MoH; MFL; VCZ
Limited molecular epidemiology data	Lack of genetic AMR surveillance limits understanding of resistance spread	Invest in laboratory capacity for molecular typing and AMR gene detection; integrate data with global platforms (FAO InFARM, WOAH ANIMUSE)	MFL; ZNPHI; MoH; FAO; WOAH

## Data Availability

The supporting data of this manuscript can be made available on request from the corresponding authors.

## Abbreviations

The following abbreviations are used in this manuscript:

AMC	Antimicrobial Consumption
AMR	Antimicrobial Resistance
AMS	Antimicrobial Stewardship
AMU	Antimicrobial Use
ANIMUSE	ANImal antiMicrobial USE global data base
FAO	Food and Agriculture Organization of the United Nations
InFARM	International FAO Antimicrobial Resistance Monitoring System
MDR	Multidrug Resistance
SADCAS	Southern African Development Community Accreditation Service
SPSS	Statistical Package for the Social Sciences
WOAH	World Organisation for Animal Health
ZNPHI	Zambia National Public Health Institute
*β*	Beta
